# An Open Access Database of Genome-wide Association Results

**DOI:** 10.1186/1471-2350-10-6

**Published:** 2009-01-22

**Authors:** Andrew D Johnson, Christopher J O'Donnell

**Affiliations:** 1 National Heart, Lung, and Blood Institute's Framingham Heart Study, Framingham, MA, USA; 2 Division of Intramural Research, National Heart, Lung and Blood Institute, Bethesda, MD, USA; 3 Cardiology Division, Department of Medicine, Massachusetts General Hospital, Boston, MA, USA

## Abstract

**Background:**

The number of genome-wide association studies (GWAS) is growing rapidly leading to the discovery and replication of many new disease loci. Combining results from multiple GWAS datasets may potentially strengthen previous conclusions and suggest new disease loci, pathways or pleiotropic genes. However, no database or centralized resource currently exists that contains anywhere near the full scope of GWAS results.

**Methods:**

We collected available results from 118 GWAS articles into a database of 56,411 significant SNP-phenotype associations and accompanying information, making this database freely available here. In doing so, we met and describe here a number of challenges to creating an open access database of GWAS results. Through preliminary analyses and characterization of available GWAS, we demonstrate the potential to gain new insights by querying a database across GWAS.

**Results:**

Using a genomic bin-based density analysis to search for highly associated regions of the genome, positive control loci (e.g., MHC loci) were detected with high sensitivity. Likewise, an analysis of highly repeated SNPs across GWAS identified replicated loci (e.g., *APOE*, *LPL*). At the same time we identified novel, highly suggestive loci for a variety of traits that did not meet genome-wide significant thresholds in prior analyses, in some cases with strong support from the primary medical genetics literature (*SLC16A7, CSMD1, OAS1*), suggesting these genes merit further study. Additional adjustment for linkage disequilibrium within most regions with a high density of GWAS associations did not materially alter our findings. Having a centralized database with standardized gene annotation also allowed us to examine the representation of functional gene categories (gene ontologies) containing one or more associations among top GWAS results. Genes relating to cell adhesion functions were highly over-represented among significant associations (p < 4.6 × 10^-14^), a finding which was not perturbed by a sensitivity analysis.

**Conclusion:**

We provide access to a full gene-annotated GWAS database which could be used for further querying, analyses or integration with other genomic information. We make a number of general observations. Of reported associated SNPs, 40% lie within the boundaries of a RefSeq gene and 68% are within 60 kb of one, indicating a bias toward gene-centricity in the findings. We found considerable heterogeneity in information available from GWAS suggesting the wider community could benefit from standardization and centralization of results reporting.

## Background

The number of genome-wide association studies (GWAS) is growing nearly exponentially, heralding an era of unprecedented discovery. Numerous novel genetic loci underlying disease susceptibility have been discovered using the unbiased GWAS approach, and many of these associations hold up to rigorous standards for replication [1]. Journal editors and scientists are increasingly calling for full disclosure of aggregate research results to accompany publication of GWAS in the form of published appendices or public websites. Under the recently implemented National Institutes of Health data-sharing policy , powerful opportunities now exist for the conduct of research using GWAS datasets due to the availability of increasing numbers of participant-level datasets. Analytic and computational approaches that further probe the results of individual studies or combine results from multiple GWAS datasets may strengthen previous conclusions, suggest novel loci or pathways [2], contribute to more calibrated effect estimates, suggest pleiotropy, refine the localization of association signals, or highlight likely functional variants [3]. A key variable for the capacity to conduct such analyses is the extent of access to full versus selective results as well as the nature and relative standardization of the information content.

While a centralized GWAS database, dbGAP, exists at NCBI, inclusion of data and results is voluntary and many GWAS have chosen not to participate, choosing instead not to release results, or to release results at a journal or independent web site [4]. A review of GWAS associations by the NHGRI has been published that grouped associations in specific disease categories [5], and a companion data table does provide a centralized resource for accessing some top GWAS results, but at the time of this submission was limited to 334 SNPs with minimal annotation (see ). The overall objective of this study was to create an open access, centralized database of significant published GWAS results, and to provide basic informatics standardization of these results in the format of the current genome build with updated gene annotations. We furthermore sought to characterize and analyze this initial GWAS database to assess data availability, data quality and annotations across all phenotypes, and to identify key genomic characteristics of GWAS associations and opportunities and obstacles to further analysis of this potentially vast genetic data space. With this objective, we collected and analyzed GWAS results compiled from a series of 118 GWAS studies published through March 1, 2008, all of which tested trait associations with > 50,000 markers, identifying genomic characteristics of associated loci in GWAS, facilitating new analyses and highlighting limitations in available data sources (study characteristics of the GWAS included are detailed [see Additional file 1]). Our initial analyses suggest novel candidate regions may be identified for further biological validation and that straightforward density analyses of associations across GWAS may be an effective way of highlighting candidate loci for further targeted analysis. Recent independent analyses have replicated genetic associations for loci suggested by our analysis (see Discussion). However, we also found reporting inconsistencies across GWAS and gaps in current reporting, suggesting substantial barriers to future analyses. To encourage further scientific cross-study exploration of published GWAS, we make our database fully available as an online supplement [see Additional file 2].

## Methods

### Collection of GWAS results in a single database

One-hundred-eighteen GWAS articles published before March 1, 2008 and their associated supplemental information was collected. The articles were identified through Pubmed searches (*GWAS, GWA, WGAS, WGA*, *genome-wide, genomewide, whole genome, all terms +association or +scan*), scanning the citations within each article and through direct searches of journal websites where GWAS were previously published. For citation information for all included articles and data sources [see Additional file 3]. All GWAS tested > 50,000 SNPs. When available via open web access, additional GWAS data was collected except if the additional data required an application process. Some papers included results for scans of multiple phenotypes or population groups. Thus, results included here reflect partial aggregate data from more than 400 individual genotype-phenotype GWAS datasets.

**Figure 1:**
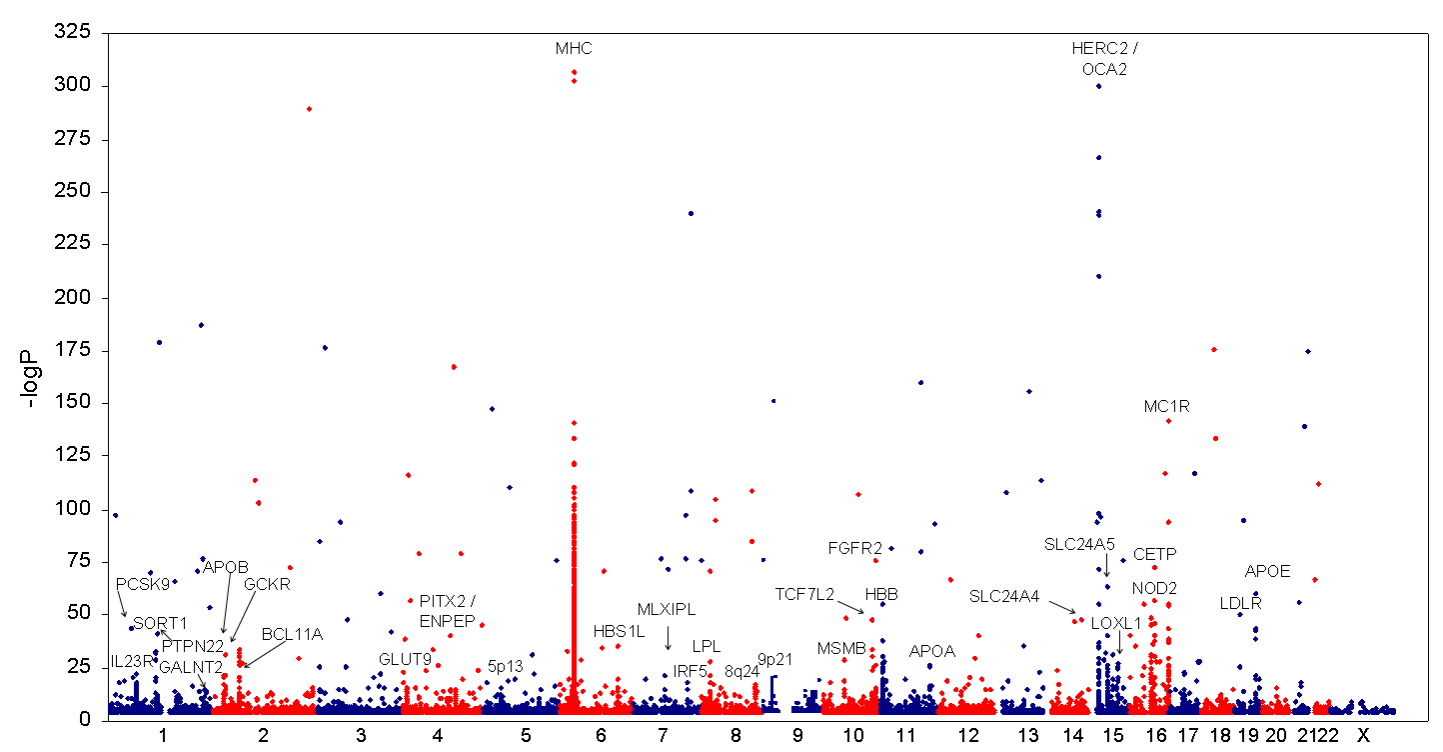
**Genome-wide plots of available GWAS results for all associations P = 0.0001**.

**Figure 2:**
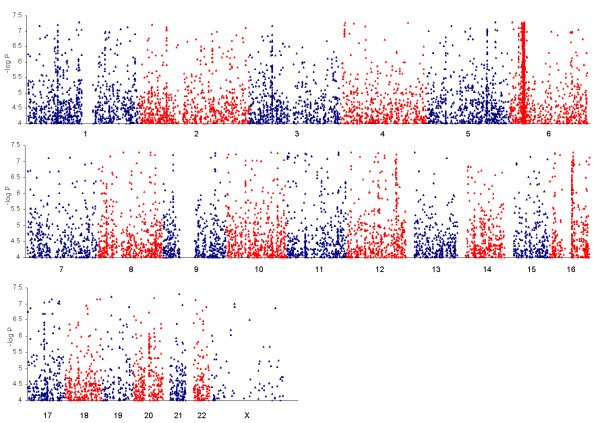
**Genome-wide plots of available GWAS results for all associations 5 × 10^-8 ^≤ P ≤ 1 × 10^-4^**.

For each article, we scanned all available text, tables, figures and accessible supplemental data to extract the most statistically significant phenotype association described in the article per SNP, meeting the following minimum criteria: 1) the SNP had an identifiable ID or verifiable genomic position, 2) a statistical p-value for association was reported, 3) the p-value was less than or equal to 0.001 (allowing for rounding) if the association was from a raw, unadjusted scan, 4) the p-value was less than or equal to 0.05 if the association was derived from replication, fine mapping or re-sequencing efforts, or if it was identified as belonging to a locus or region that was specifically identified as an *a priori *candidate by the authors. In many cases due to the large amount of available data or the non-uniform data format, a custom Perl program was written to facilitate the processing of the associations. We did not collect full disclosure association results for scans with density < 200,000 SNPs, even though these full disclosure association results were available in some cases. The primary reason for this analytic decision was that the wide availability of many trait results for lower density scans would result in an extremely large meta-dataset biased toward lower density genotyping results which have less power to detect true associations. Likewise, the discovery scan p-value threshold was set to p < 0.001 to create a set of significant GWAS results of manageable size in which the representation of significant results from studies that released limited results would not be dwarfed by results from studies that released most, or all, results. Information specific to each GWAS and to each SNP-phenotype meeting our criteria was collected in a single table [see Additional file 2]. This table represents a large, open access database of GWAS results (also presented as a Microsoft Access database [see Additional file 4]). For an extended description of each data field and how they were derived [see Additional file 3]. Genome-wide plots of all included associations are shown in Figure 1, and for those associations above the threshold of 5 × 10^-8 ^in Figure 2.

We independently verified the quality of the extracted GWAS results database in a blinded fashion. Three independent reviewers extracted information from the same 2 GWAS articles in parallel to ensure they applied our inclusion criteria in the same manner. Twelve of the 118 GWAS articles were randomly selected. These articles were assigned 4 each to 3 reviewers. Each reviewer independently generated information from the GWAS article according to the guidelines above and then compared their results to the original extracted results.

### Incorporation of current genomic annotations

Information from the GWAS papers spanned at least 3 human genome Builds and 12 dbSNP builds, resulting in SNP positions and SNPids that have shifted in some cases. Additionally, some papers gave only genome coordinates without SNPids or supplied only commercial chip IDs. Thus, in order to maximize the analysis of available GWAS results in the current genome build context and retrieve current SNP annotation it was necessary to apply multiple strategies to update SNP coordinates and SNPids. For some markers old genome coordinates were translated into current coordinates using the UCSC Genome Browser LiftOver conversion tool in order to discover missing SNPids. When only commercial chip IDs were given these were translated into rsIDs using the most appropriate annotation files from the corresponding company. For some associations we were unable to establish SNP identification based on the information provided in the original report. Although this was only the case for a handful of associations, it does suggest more vigilance is required by journals in order to standardize the reporting of genetic variants (e.g., SNP identifiers and precise genome coordinates).

To facilitate retrieval of current SNP annotation information we wrote a Perl program, GRASP (Genome-wide Retrieval of Annotation for SNPs Program, available upon request). Current coordinates were retrieved from the dbSNP table "b128_SNPChrPosOnRef_36_2". Alias SNP IDs were retrieved from the "RsMerge128Arch" table and used to find current coordinates when necessary. SNPs that mapped to multiple genome locations were noted and further gene annotation was not included for these. The GRASP program integrated UCSC human genome browser annotation tracks for RefSeq genes and UCSC Known genes, yielding standardized annotations for overlapping and nearby genes for all GWAS SNP associations [see Additional file 2].

### Characterization and analysis of a GWAS results database

The main GWAS database contains 56,411 unique SNP-phenotype entries [see Additional file 2]. The database represents results from a heterogeneous set of studies with varied amounts and types of data available. Thus, we did not attempt to conduct formal statistical meta-analyses. Rather, our primary aim was to use this database to make observations that either strengthen prior associations or highlight them in a new way (e.g., in relation to additional phenotypes), or are suggestive of regions for future investigations. Using Perl programs, we: 1) enumerated and ranked repeated occurrences of individual SNPs across GWAS studies, 2) split the genome into 100 kb bins and counted SNP-phenotype associations within each bin, and 3) determined the average pairwise LD within each 100 kb bin based on the HapMap CEU data (release #23a). After standardizing the gene annotations for all associations, we applied High-Throughput GOminer analysis software to search for gene ontologies that are statistically over-represented among significant GWAS associations [6]. For this approach SNP associations directly within genes nominated those genes as positively associated with a given trait or set of traits. GOminer tests for the over-enrichment of gene ontologies in large gene sets, using an FDR approach based on repeated random sampling to account for multiple testing [6]. To test the sensitivity of the gene ontology findings to the inclusion of specific data we ran further analyses without the WTCCC and DGI results, and within specific disease subsets ([see Additional file 3] for a more detailed description of the approach and the disease subsets).

## Results

### Marked heterogeneity in completeness and annotation of reported GWAS results

Full disclosure genotype-phenotype association results were publicly available for every SNP tested for only a minority of the GWAS scans. In 45% (n = 53) of GWAS articles, fewer than 40 SNP-specific association results were made publicly available, and in many studies results for very few loci and SNPs were disclosed (25 studies reported results for 10 or fewer SNPs). In thirty-one (26%) articles, the authors disclosed the complete set of associations, and in the remaining articles (n = 34), they disclosed only a moderate number of top-ranked associations (defined as ≥ 40 associations for ≥ 4 distinct loci). There was also substantial heterogeneity in the format and type of results data available from GWAS studies. In many studies, information regarding SNP strand, alleles and direction of effect, sample sizes passing quality control for individual SNPs, and genetic model were unavailable, thus precluding or limiting the conduct of formal meta-analyses. Despite the heterogeneity and limited data availability, we extracted a minimal redundancy database of 56,411 statistically significant SNP-phenotype associations across all studies by use of custom computer programming to facilitate further analysis. Briefly, the criteria for inclusion was the most significant mention per SNP per study, and only included SNPs with unadjusted genome-wide p-values for association ≤ 1 × 10^-3^, or which were significant in replication or further analysis at p = 0.05. (A full description of criteria for inclusion is found in the Methods, for full results [see Additional file 2]). We validated the completeness and accuracy of the extracted SNP database by a re-extraction of a random selection of 10% of the studies conducted by a panel of three reviewers. We found no detectable errors in regards to the total number and identity of SNPs that were included in the final dataset.

### Informatics challenges arising from currently reported GWAS results

**Figure 3:**
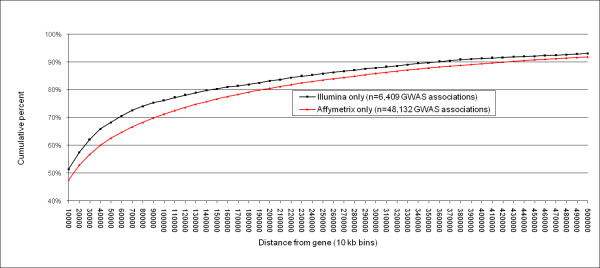
**Cumulative percent of GWAS association SNPs falling within specified distances from a protein-coding RefSeq gene**. SNP frequency by distance is plotted for significant GWAS associations from studies that employed only Illumina of only Affymetrix platforms (in 10 kb bins).

**Figure 4:**
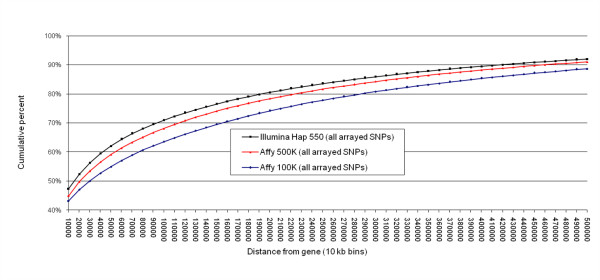
**Cumulative percent of arrayed SNPs falling within specified distances from a protein-coding RefSeq gene**. SNP frequency by distance is plotted for arrayed SNPs on Affymetrix or Illumina arrays (in 10 kb bins). The gene-centric arrays from each company are not displayed since relatively few studies analyzed here have published results based on these arrays. The Illumina Hap240, Hap300 and Hap650 are omitted because they show a highly similar pattern to the Hap550 array.

Currently available GWAS results span 3 builds of the human genome (34–36) and at least 12 builds of dbSNP (118–129). Since SNPids are being modified and merged over time, and the relative positions of SNPs often shift between human genome builds, there are substantial informatics challenges to GWAS meta-data accumulation, analysis and viewing. Using current dbSNP information, including mapping of alias SNPids, we migrated all reported SNPs from GWAS associations in Additional file 2 into the current framework of human genome Build 36.2 positions. Relying on these positions, we then re-annotated all associations with protein-coding gene information (see Methods) and compared current annotations with those originally described in GWAS results reports. In contrast to the original annotations from the GWAS articles and datasets, in which 23.3% of associated SNPs were reported to be in or near genes ([see Additional file 2], column V), when we applied standardized annotation we found 40.0% of associated SNPs are within the transcript boundaries of a RefSeq gene, indicating a relative under-estimation of the proximity to genes of loci in initial GWAS reports. Furthermore, from our database, we found that most top GWAS associations are relatively gene-centric, with 65.7% of the associated SNPs located either in or within 60 kb of a RefSeq gene (Figure 3). Significantly associated SNPs showed a trend toward being more gene-centric than all SNPs present on the arrays used in most studies (seen in the contrast between Figure 3 and Figure 4). We compared studies that employed either Affymetrix only or Illumina only arrays and we found little difference in the proportion of associated SNPs located within genes (Figure 3, Affymetrix: 39.5%, Illumina: 40.8%). When we considered associated SNPs in or near (within 60 kb) a RefSeq gene, there were a modestly increased proportion of gene centric associations within "Illumina only" studies (Figure 3, Affymetrix: 64.7%, Illumina: 70.6%).

### First analysis of a cross-GWAS results database reveals SNPs associated with two or more diverse phenotypes

Using our standardized GWAS results database [see Additional file 2], we conducted a density analysis to find the densest regions of association in the genome, using 100 kb bins across the genome and including all SNP associations regardless of the magnitude of statistical signals. To account for LD, which could confound our analysis by inflating the density of associations in regions of high LD, we ran a parallel analysis where we adjusted for the average pairwise LD (r^2^) in the same regions for the HapMap CEU samples. Both analyses identified many previous strongly replicated loci within regions of the genome showing the highest density of previous associations (Table 1). In Figure 1, we provide a view of GWAS associations across a diverse set of phenotypes, including many regions of the genome where SNP associations exceed the common genome-wide significance threshold (*P *< 5 × 10^-8^), clearly highlighting both the density and magnitude of association signals at many replicated loci. Figure 2 shows a restricted view of GWAS results from 5 × 10^-8 ^≤ P ≤ 1 × 10^-4^, making apparent a number of clusters that approach the genome-wide significant threshold (*P *< 5 × 10^-8^). Across the genome, the 99^th ^percentile cutpoint based on density of associations included bins with 13 or more GWAS associations within less than or equal to 100 kb. The MHC class II loci contained the densest bins. There are numerous phenotypes associated with the MHC loci, consistent with significant pleiotropy of this region, but there is also evidence for pleiotropy for a previously replicated Alzheimer's disease locus (*MAPT*, *KIAA1267*, *STH*), which displays a signal for Crohn's disease, and a second replicated Alzheimer's locus (*DAPK1*), which shows evidence for Type II diabetes and related traits across multiple studies.

**Table 1:** Densest regions of GWAS associations in the human genome based on analysis of 100 kb bins.

Chr	Position	GWAS assoc. in bin	Density rank	LD adj. rank	Min. p value	Gene(s)	Phenotype(s) in decreasing order of occurrence
6	32405288–32497626	177	1	1	5.47E-111	*C6orf10, BTNL2*	RA (65), Type I (50), MS (26), Type II (17), combined WTCCC cases (10), CAD (8), Alzheimer's (1)
6	32703038–32798923	107	3	2	1.12E-307	*HLA-DQA1, HLA-DQB1*	Type I (47), RA (30), MS (14), combined WTCCC cases (7), Celiac disease (3), SLE (2), Crohn's disease (1), AAT levels (1), Type II (1), Alzheimer's (1)
6	32806435–32896489	116	2	3	4.86E-79	*HLA-DQA1, HLA-DQB1, HLA-DOB*	Type I (59), RA (25), MS (21), combined WTCCC cases (9), Early onset obesity (1), 2 hr glucose tolerance test (1)
2	60502647–60591731	77	5	4	6.70E-35	*BCL11A*	HbF (75), Type II (1), Hypertension (1)
2	60415435–60494833	87	4	5	2.50E-12	downstream from *BCL11A*	HbF (75), Type II (7), CAD (1), Abdominal aortic calcification (1), combined WTCCC cases (1), Bipolar (1), Parkinson's (1)
1	67406551–67498692	65	6	6	3.03E-23	*IL23R*	Crohn's disease (60), Psoriasis (2), RA (1), CAD (1), combined WTCCC cases (1)
9	89310993–89384465	46	9	7	2.80E-05	*DAPK1*	Alzheimer's (19), HOMA-IR (17), Waist:height (5), Type II (2), Fasting insulin (2), RA (1)
6	151254904–151296407	17	91	8	2.90E-08	*MTHFDL1*	CAD (10), Type 1 (2), combined WTCCC cases (1), Hypertension (1), FPG (1), Insulinogenic index (1), ALS (1)
11	5401462–5499595	29	23	9	9.46E-24	*HBB, UBQLN3*, olfactory receptors	MCV (8), HbA2 (7), RBC (3), HbA1C (1), CAD (3), Type II (3), Fat mass (2), Bipolar disorder (1), Crohn's disease (1)
16	55526649–55589312	22	43	10	1.00E-73	*CETP*	HDL cholesterol (16), Type II (3), ApoAI (2), HTN (1)
9	116701486–116796674	31	22	11	1.61E-09	*TNFSF8, TNC*	Crohn's disease (29), 2 hr glucose tolerance test (2)
8	128116770–128194686	24	31	12	1.10E-12	intergenic, 8q24	Colorectal cancer (10), Prostate cancer (8), Bipolar disorder (3), CAD (2), Hypertension (1)
8	19857092–19899552	42	11	13	2.00E-28	*LPL*	Triglycerides (22), HDL (11), Triglyceride:HDL (4), CAD (4), RA (1), Hip circumference (1)
9	22102599–22126489	26	29	20	1.00E-20	*ANRIL*, 3' of *CDKN2A/2B*	Type II (12), CAD (11), combined WTCCC cases (2), Coronary artery calcification (1)
10	123321680–123395011	28	24	21	2.00E-76	*FGFR2*	Breast cancer (24), RA (2), Type II (1), combined WTCCC cases (1)
6	20706282–20796100	33	21	28	4.11E-11	*CDKAL1*	Type II (32), Insulin response (1)
16	49300832–49399578	47	8	31	2.51E-49	*NOD2*	Crohn's disease (44), RA (1), combined WTCCC cases (1), ApoB (1)
16	52327178–52389272	38	14	33	7.30E-14	*FTO*	TypeII (15), Hip circumference (15), Early onset obesity (6), Fasting glucose (1), ALS (1)
10	114722872–114798892	39	13	34	1.00E-48	*TCF7L2*	Type II (36), Waist:height (2), BMI (1)
9	22007836–22093813	52	7	41	2.10E-19	*MTAP, ANRIL, CDKN2A/2B*	CAD (48), Coronary artery calcification (2), Type II (2), Type I (1), RA (1)
17	35304874–35382291	44	10	43	2.00E-23	*ORMDL3*	Childhood asthma (34), ORMDL3 expression (7), Crohn's disease (3)
1	228303905–228375529	24	32	55	7.00E-15	*GALNT2*	Type II (9), Triglycerides (7), HDL (6), Crohn's disease (1), ALS (1)
16	49218265–49298963	34	19	73	4.71E-29	*NOD2, SLIC1, CYLD*	Crohn's disease (33), Bipolar disorder (1)
11	41805501–41887387	34	20	74	5.70E-08	11p12, 800 kb from *NGL1*	Type II (33), Coronary spasm (1)
1	109608806–109623689	23	37	92	1.20E-33	*SORT1, CELSR2, PSRC1, MYBPHL, SARS*	LDL cholesterol (16), CAD (4), Total cholesterol (1), Bipolar disorder (1)
8	128404855–128498005	23	38	99	1.27E-14	*POU5F1*	Prostate cancer (8), Colorectal cancer (13), Breast cancer (1), Hypertension (1)
5	40513272–40597115	40	12	264	8.73E-12	intergenic, 5p31.1	Crohn's disease (40)
20	33403387–33489397	36	17	322	5.01E-12	*GDF5, UQCC, CEP250*	Height (35), Hypertension (1)
10	64100271–64194296	24	35	494	1.00E-10	*ZNF365, ADO, EGR2*	Crohn's disease (16), Type II (8)
17	41401810–41495480	38	15	526	3.53E-05	*KIAA1267, MAPT, STH*	Crohn's disease (26), Alzheimer's (11), Insulinogenic index (1)
10	44011247–44096316	23	39	647	9.46E-08	intergenic, 10q11.21	CAD (20), Diastolic blood pressure (2), Fasting glucose (1)
17	41305238–41395352	37	16	746	2.78E-05	*KIAA1267, MAPT, STH*	Crohn's disease (19), Alzheimer's (18)
2	21223320–21252721	27	27	796	8.10E-09	upstream of *APOB*	LDL cholesterol (17), Total cholesterol (10)

Densest regions of GWAS associations in the human genome previously highlighted in a GWAS study (based on analysis of 100 kb bins). Bin rankings are given based on simple counts of associations, and after adjusting for HapMap CEU LD (average pairwise r^2 ^in the same region), out of 18,310 bins in the genome that contained 1 or more GWAS association. Bins with known, replicated associations ranked in the top 40 by density and/or after LD adjustment are listed.

We considered regions that were not highlighted in the original GWAS articles but that nonetheless reveal a high density of associations in our analysis (Table 2). Although associations were noted in more than two studies for all of these regions, none of the single SNP associations was considered to be significant on a genome-wide level. A dense cluster of significant associations for Crohn's disease and HDL cholesterol is located in the monocarboxylate transporter 2 gene (*SLC16A7*, also known as *MCT2*), a ubiquitously expressed transporter that imports and exports lactate and pyruvate. Other clusters of interest include: a complement related factor, *CSMD1*, mainly for association with HIV-1 viral load; and associations with Crohn's disease and Type I diabetes at *OAS1*, an enzyme that degrades viral RNA and has previously been associated with Type I diabetes, multiple sclerosis and SARS infection. Accounting for LD in the density analysis changed the rankings of the top regions (Tables 1 and 2), but all of the unadjusted bins remained within the top 5% of all bins for known, replicated regions (Table 1) and within the top 10% of all bins for those presented in Table 2.

**Table 2:** Densest regions of GWAS associations in the human genome based on analysis of 100 kb bins.

Chr	Position	GWAS assoc. in bin	Density rank	LD adj. rank	Min. p value	Gene(s)	Phenotype(s) in decreasing order of occurrence
10	6316580–6390596	17	92	14	2.70E-06	*PFKFB3*	LDL cholesterol (4), Total cholesterol (4), Celiac disease (2), RA (2), Waist circumference (2), Bipolar disorder (2), Weight (1)
13	109616103–109677555	19	67	15	7.61E-06	*COL4A1*	Weight (11), Type II (2), ALS (2), BMI (1), Height (1), Diastolic blood pressure (1), combined WTCCC cases (1)
8	4621850–4669277	27	25	16	1.12E-04	*CSMD1*	HIV-1 viral load set point (25), Crohn's disease (1), combined WTCCC cases (1)
3	21847197–21874359	20	56	17	2.00E-05	intergenic, 3p24.1	Type II (10), Diastolic blood pressure (4), Bipolar disorder (2), Alzheimer's (2), Parkinson's (1), Crohn's disease (1), combined WTCCC cases (1)
13	23522469–23566520	15	142	18	2.62E-04	*SPATA13*	Fat mass (8), Type II (3), Alzheimer's (2), Crohn's (1), RA (1)
13	109706778–109769126	9	447	24	2.0E-05	*COL4A1*	HbA1c (3), CAD (2), RA (1), ApoB (1), Crohn's (1), Parkinson's (1), Alzheimer's (1)
20	59002911–59076486	17	94	25	2.11E-05	intergenic, 20q13.33	CAD (6), Type II (2), combined WTCCC cases (2), HTN (2), RA (1), Crohn's disease (1), Breast cancer (1), Alzheimer's (1), ALS (1)
2	121639597–121685155	15	143	26	4.00E-06	intergenic, 2q14.2	Alzheimer's (4), Triglyceride/HDL ratio (4), Triglycerides (3), HDL (1), Type I (1), RA (1), Insulinogenic index (1)
4	178511559–178569631	10	448	30	6.90E-06	*NEIL3*	ALS (3), Parkinson's (1), Bipolar disorder (1), Type II (1), Type I (1), combined WTCCC cases (1), mFPG (1), heart rate variability (1)
16	76400711–76498035	11	333	32	1.10E-04	*KIAA1576*	2 hr insulin test (5), combined WTCCC cases (2), LV diastolic dimension (1), Fasting insulin (1), HOMA-IR (1), Alzheimer's (1)
3	2801545–2885980	11	334	35	2.48E-05	*CNTN4*	Total cholesterol (3), HIV-1 viral load set point (2), combined WTCCC cases (1), RA (1), Waist:height squared (1), 2 hr glucose tolerance test (1), Alzheimer's (1), ALS (1)
22	48109439–48199236	9	583	42	3.76E-05	intergenic, 22q13.32	Type II (3), CAD (2), HTN (2), LDL (1), Small vessel stroke (1)
7	77505244–77596871	17	95	44	1.38E-04	*MAGI2*	BMI (5), Waist circumference (5), Waist:height squared (5), HTN (1), ALS (1)
16	6603645–6620427	15	145	45	2.31E-05	*A2BP1*	HOMA-IR (6), Fasting insulin (4), 2 hr insulin test (3), RA (1), Nicotine dependence (1)
8	134608378–134674046	14	177	46	5.66E-05	*ST3GAL1*	Type II (6), Alzheimer's (6), Crohn's disease (2)
11	113445679–113499715	13	221	47	1.25E-05	*ZBTB16*	Waist circumference (4), HDL (3), BMI (2), Weight (2), Type II (1), LDL (1)
8	4200701–4270026	10	450	51	2.79E-04	*CSMD1*	HIV-1 (4), HTN (1), Systolic blood pressure (1), Type I (1), FPG (1), Colorectal cancer (1), Alzheimer's (1)
20	54803001–54891697	8	761	52	2.65E-04	intergenic, 20q13.31	ApoB (2), CAD (1), HTN (1), Fasting glucose (1), 2 hr glucose (1), Alzheimer's (1), Triglyceride/HDL ratio (1)
13	109813629–109898019	15	146	53	1.89E-05	*COL4A2*	Type II (10), CAD (2), HTN (1), ALS (1), Alzheimer's (1)
16	82802796–82891261	8	762	54	1.60E-05	*KCNG4, WFDC1*	HIV-1 (2), Type II (1), Crohn's disease (1), Fat mass (1), Insulinogenic index (1), ALS (1), combined WTCCC cases (1)
6	164313234–164367157	22	44	135	3.84E-05	intergenic, 6q26	Insulinogenic index (14), LDL (3), HOMA-IR (2), Alzheimer's (1), Parkinson's (1), RA (1)
13	63720215–63799016	24	33	148	7.46E-05	intergenic, 13q21.31	HDL cholesterol (8), Waist circumference (7), Weight (5), CAD (2), BMI (1), Gallstone disease (1)
3	162876798–162898990	24	34	229	2.41E-05	intergenic, 3q26.1	BMI (7), Waist circumference (6), Waist:height (6), Weight (2), Early onset Type II (1), Hypertension (1), Crohn's disease (1)
12	58400177–58492903	35	18	267	1.12E-05	*SLC16A7*	Crohn's disease (21), HDL (14)
21	40500455–40546492	22	46	326	9.05E-05	*DSCAM*	Height (11), Weight (7), Waist circumference (4)
12	111841825–111894699	22	47	440	5.92E-05	*OAS1/2/3, RPH3A*	Crohn's disease (18), Type I (4)
7	86415282–86498669	23	40	1434	1.60E-04	*KIAA1324L*	Type II (20), Triglyceride/HDL ratio (2), CAD (1)
4	35101778–35198927	23	41	1569	7.72E-05	intergenic, 4p15.1	Nicotine dependence (21), Type I (1), Hypertension (1)

Densest regions of GWAS associations in the human genome not previously highlighted in a GWAS study (based on analysis of 100 kb bins). Bin rankings are given based on simple counts of associations, and after adjusting for HapMap CEU LD (average pairwise r^2 ^in the same region), out of 18,310 bins in the genome that contained 1 or more GWAS association. Bins with associations not previously highlighted and ranked in the top 55 by density and/or after LD adjustment are listed.

### Highly repeated SNPs across GWAS association results

After accounting for aliases used for identical SNPs, we counted the frequency of occurrence of individual SNPs among top GWAS associations to search for SNPs associated repeatedly across traits. Among all SNP associations (n = 56,411), 52,554 unique SNPs were observed, and the bulk of these SNPs were associated with a single phenotype once (n = 49,313). Examining the most redundant SNPs across GWAS associations revealed a set of known, replicated loci, validating this approach (Table 3). For example, a single SNP located 3' of *APOC1 *(rs4420638) was associated 11 times across GWAS, including association with Alzheimer's disease, lipid-related traits and coronary artery disease (CAD). Some replicated loci contained multiple SNPs with repeated associations, which may be due to LD and differences in representation on arrays (*APOB*, *LPL*, *TCF7L2*, *SORT1, CELSR2, PSRC1*, and *FTO *in Table 3). By searching for repeated SNP associations, a number of new suggestive loci were also observed, each of which was associated independently with five traits, with none reaching genome-wide significance. These included: *GABRG3 *(rs968671) which showed association with BMI-related traits and hypertension and is located in a cluster of GABA receptor subunits, notable for the role of GABA signaling in sympathetic vasomotor tone; *RAPGEF1 *(rs7034356, rs4740294), an exchange factor involved in cell signaling, was associated with BMI-related traits; *PIGU *(rs2889849), a subunit of glycosylphosphatidylinositol transamidase, was associated with lipid- and BMI-related traits; and *SPAG16 *(rs10498015), a sperm-associated protein, was associated with height, weight and lipid-traits (Table 3).

**Table 3:** Top repeated occurrences from 52,554 unique SNPs among 56,411 SNP-phenotype associations.

SNPid	Repeat assoc.	Gene(s)	Trait(s)	P value range	Chr	Position	Gene size	SNP on platforms?	Perfect CEU LD proxies on other platforms?
rs4420638	11	*APOE/APOC *cluster	Lipids, Alzheimer's, CAD	1.7E-4 to 1.0E-60	19	50114786	4 kb	Affymetrix, Illumina	Y
rs599839	8	*PSRC1 *(near *CELSR2, SORT1*)	Lipids, CAD	2.2E-5 to 1.2E-33	1	109623689	88 kb	Affymetrix, Illumina	Y
rs693	8	*APOB*	Lipids	6.8E-4 to 1.0E-21	2	21085700	43 kb	Affymetrix, Illumina	Y
rs17482753	6	*LPL*	Lipids	3.6E-5 to 5.9E-19	8	19876926	29 kb	Affymetrix	Y
rs780094	6	*GCKR*	Triglycerides, Crohn's	6.0E-3 to 1.0E-15	2	27594741	27 kb	Affymetrix, Illumina	Y
rs1801282	6	*PPARG*	TypeII, Alzheimer's, Obesity	4.5E-2 to 1.7E-6	3	12368125	146 kb	Affymetrix, Illumina	Y
rs910049	5	MHC locus (*C6orf10*)	TypeII, TypeI, MS, CAD	7.8E-4 to 3.0E-93	6	32423705	79 kb	Affymetrix, Illumina	Y
rs2076530	5	MHC locus (*BTNL2*)	MS, TypeI, RA	3.2E-10 to 6.7E-74	6	32471794	12 kb	Affymetrix, Illumina	Y
rs7901695	5	*TCF7L2*	TypeII, Waist:Height	2.6E-4 to 1.0E-48	10	114744078	216 kb	Affymetrix, Illumina	Y
rs7903146	5	*TCF7L2*	TypeII	5.5E-8 to 1.0E-48	10	114748339	216 kb	Illumina	N
rs4506565	5	*TCF7L2*	TypeII, BMI	8.6E-4 to 2.3E-31	10	114746031	216 kb	Affymetrix	N
rs10503669	5	*LPL*	Lipids	9.1E-4 to 3.9E-22	8	19891970	29 kb	Affymetrix	Y
rs562338	5	*APOB*	Lipids	2.2E-6 to 5.6E-22	2	21141826	43 kb	Affymetrix	Y
rs11209026	5	*IL23R*	Crohn's, Psoriasis	4.8E-4 to 6.6E-19	1	67478546	93 kb	Affymetrix, Illumina	Y
rs8050136	5	*FTO*	BMI, TypeII	9.8E-7 to 7.3E-14	16	52373776	410 kb	Affymetrix, Illumina	Y
rs9939609	5	*FTO*	BMI, TypeII	1.9E-6 to 9.0E-12	16	52378028	410 kb	Affymetrix	Y
rs4970834	5	*CELSR2 *(near *SORT1, PSRC1*)	Lipids, CAD	3.4E-4 to 3.0E-11	1	109616403	26 kb	Affymetrix, Illumina	Y
rs1111875	5	*HHEX*	TypeII	1.4E-3 to 5.7E-10	10	94452862	6 kb	Affymetrix, Illumina	Y
rs13266634	5	*SLC30A8*	TypeII	3.3E-6 to 5.3E-8	8	118253964	42 kb	Illumina	N
rs968671	5	*GABRG3*	BMI, Hypertension	3.3E-4 to 2.5E-6	15	25036654	650 kb	Affymetrix	N
rs7034356	5	*RAPGEF1*	BMI	3.7E-4 to 1.3E-5	9	133468838	161 kb	Affymetrix	Y
rs4740294	5	*RAPGEF1*	BMI	8.3E-4 to 1.3E-5	9	133462842	161 kb	Affymetrix	Y
rs481843	5	Intergenic (near *APO *cluster, *BUD13*)	Lipids, Alzheimer's	9.3E-4 to 2.1E-5	11	116031077	-	Affymetrix	Y
rs2889849	5	*PIGU*	BMI, Lipids	8.8E-4 to 1.9E-4	20	32627938	117 kb	Affymetrix, Illumina	Y
rs10498015	5	*SPAG16*	Lipids, Hgt, Wgt	9.7E-4 to 1.2E-4	2	214425824	165 kb	Affymetrix, Illumina	Y

Highly repeated occurrences of unique SNPs among 56,411 SNP-phenotype associations. We report the presence of these SNPs and whether a perfect proxy LD SNP exists based on the HapMap CEU population, on both Affymetrix and Illumina platforms, and the other platform in the case of a SNP only genotyped on an array from one company. This information was determined using the SNAP tool [33] and demonstrates that signals from a small minority of these SNPs could be missed depending on the platform applied in each case. Underlined entries represent newly recognized loci or phenotype associations.

### Over-represented functional gene categories among top GWAS association results

Using our standardized RefSeq gene annotations of GWAS associations, we identified all protein-coding genes containing one or more association among top GWAS results (n = 5,966). We explored whether genes with specific types of biological function are over-represented across significant GWAS results using GOminer, software originally designed for microarray analysis [6]. Genes relating to cell adhesion functions were highly over-represented (*P *< 4.6 × 10^-14^) across the meta-dataset, as were genes related to signal transduction (*P *< 9.7 × 10^-11^), transport activity (*P *< 1.1 × 10^-9^), and protein phosphorylation (*P *< 2.4 × 10^-7^) (Table 4). To test the sensitivity of these findings to the inclusion of specific datasets, we repeated the analysis after removing data from two of the largest data contributors (WTCCC, DGI) leaving a subset of associations within 2,888 genes. In the repeat analysis, the distribution and statistical significance of associations among the top biological function categories was not significantly altered (Table 4). In an analysis stratified by major disease categories ([see Additional file 3] for the specific studies included in each set), we found that genes relating to cell adhesion were significantly over-represented in every disease set and ion transport related genes were significantly over-represented in every disease set except lipid-related traits [see Additional file 5]. Examining significantly associated protein-coding gene categories with FDR < 0.05 in each disease set revealed a positive control for this approach, the "antigen processing and presentation" gene category in the rheumatoid arthritis set (*P *< 2.4 × 10^-9^) [see Additional file 5]. A number of other over-represented categories were also concordant with the expected specific disease contexts: "nervous system development" (ALS, Alzheimer's disease, Weight/BMI), "synaptic transmission" (ALS), "metal ion/sodium/calcium transport" (CAD, Hypertension), "phospholipid transport" (Type II Diabetes), and "response to nutrient levels" (Weight/BMI).

**Table 4:** GOminer gene ontology analysis of GWAS results, indicating the most over-represented ontological categories.

	Including WTCCC/DGI results (n = 5966 genes)					Excluding WTCCC/DGI results (n = 2888 genes)
Gene Ontology category	RefSeq genes in Category	RefSeq genes observed in GWAS	GO category enrichment	P-value for enrichment	FDR	P-value for enrichment

GO:0007155_cell_adhesion	650	309	1.41	4.56E-14	0	1.99E-17
GO:0051056_regulation_of_small_GTPase_mediated_signal_transduction	161	94	1.73	9.73E-11	0	3.76E-06
GO:0051179_localization	2493	975	1.16	1.12E-10	0	2.32E-07
GO:0006810_transport	2128	837	1.17	1.13E-09	0	2.46E-06
GO:0051234_establishment_of_localization	2189	856	1.16	2.64E-09	0	2.92E-06
GO:0006811_ion_transport	682	297	1.29	2.39E-08	0	1.32E-08
GO:0009966_regulation_of_signal_transduction	408	189	1.37	5.37E-08	0	0.00132
GO:0046578_regulation_of_Ras_protein_signal_transduction	122	70	1.70	6.27E-08	0	0.00014
GO:0007156_homophilic_cell_adhesion	134	75	1.66	8.92E-08	0	6.35E-09
GO:0016337_cell-cell_adhesion	245	121	1.46	2.26E-07	0.000091	1.04E-07
GO:0006468_protein_amino_acid_phosphorylation	536	236	1.31	2.43E-07	0.000083	7.42E-07
GO:0043687_post-translational_protein_modification	1171	473	1.20	2.83E-07	0.000154	0.00097
GO:0007265_Ras_protein_signal_transduction	177	92	1.54	3.59E-07	0.000143	0.00013
GO:0006464_protein_modification_process	1399	555	1.18	3.91E-07	0.0002	0.00721
GO:0007242_intracellular_signaling_cascade	1210	486	1.19	4.26E-07	0.000188	0.00040
GO:0006817_phosphate_transport	83	50	1.79	6.31E-07	0.000176	2.10E-05

## Discussion

In our evaluation of a comprehensive GWAS results database across diverse phenotypes, we confirm the potential benefit of open access to GWAS results data by a series of observations. After re-annotation of all reported results, we determined that more than two-thirds of associations are in or within reasonably limited physical and genetic distance from a protein-coding gene, with a significant minority of associations more distant from a protein-coding gene. While intentionally hypothesis generating, the results of our analyses (Tables 1, 2, 3, 4, and [see Additional file 5]) suggest there are a number of novel associated loci, pleiotropic effects of known loci, and newly emphasized functional gene categories in human diseases. Using standardized gene annotations of top GWAS associations, we further undertook an ontology-based functional analysis, revealing a striking over-representation of cell adhesion-related genes implicated in GWAS studies encompassing a diversity of diseases (*P *< 4.6 × 10^-14 ^for all diseases). We make the compiled results fully available in supplemental files, [see Additional file 2] or [see Additional file 4], and also provide input files that can be used to visualize all associations included here, or from specific studies, using UCSC Genome Graphs [see Additional file 6].

Using a straightforward bin clustering analysis of all GWAS results we identified known, replicated loci, but also observed high density clustering of associations in gene regions that were not previously highlighted in the primary GWAS studies, but displayed significance in two or more GWAS (Table 2). The densest cluster of such associations was observed for Crohn's disease and HDL cholesterol in the 3' region of a monocarboxylate transporter, *SLC16A7*, also known as *MCT2*. Notably a related monocarboxylate transporter, *MCT1*, was shown to be decreased in expression in the inflamed colonic mucosa of patients with ulcerative colitis and Crohn's disease relative to controls [7]. The next densest cluster was primarily associated with HIV-1 viral set point [8] in *CSMD1*, a gene which encodes a soluble protein that can block the classical complement activation pathway [9]. This is of particular interest since a characteristic of HIV-1 infection and persistence is the active evasion of the host humoral response, a key component of which is complement activation [10].

The preceding examples, and others in Tables 2 and 3 (*RAPGEF1*, *PIGU*, *SPAG16, PFKFB3, COL4A1/2, A2BP1*) suggest novel candidate genetic loci that require further replication, but we also noted GWAS associations of interest in at least one locus with previous evidence for association. A gene encoding 2',5' oligoadenylate synthetase 1 (*OAS1*) is stimulated by interferon, plays an important role in innate immunity and was previously shown to be genetically associated with Type I Diabetes [11], multiple sclerosis [12], SARS [13] and hepatitis C persistent infection [14]. Here we report signals in GWAS results for both Type I Diabetes and Crohn's disease which, given prior associations, suggests this locus may harbor at least one functional allele that impacts a range of immune-related etiologies. Arguably, this example may demonstrate that previous candidate gene centered associations can be replicated via *in silico *analysis of GWAS results. During the review of this article, published and unpublished studies came to our attention, which provide some additional validation for results we present. We noted in Table 3 the highly repeated association of SNPs in genes including *RAPGEF1 *and *PIGU *across multiple GWAS and suggested these as potential novel candidate genes for further study and replication. Recently a genome-scan for melanoma, reported the most significant association, which was replicated, was found in *PIGU *(p < 1.0 × 10^-15^) [15]. The genome-wide significant SNP from Brown and colleagues is in significant LD with the SNP present in Table 3 (D' 1.0, r^2 ^0.57). This genomic region (20q11.22) also ranked relatively high in our bin-based analysis (density rank = 78, LD-adjusted rank = 1,338) as a previously, unreplicated region that contained a high density of GWAS associations for diverse diseases. In an analysis of 222 candidate genes for association with diabetes and related traits, extending previously published GWAS analyses, Gaulton and colleagues [16] report a *RAPGEF1 *SNP (rs4740283) as the most statistically significant associated SNP with Type II Diabetes among all SNPs and genes they analyzed. This SNP is nearby and in complete LD (D' 1.0, r^2 ^1.0) with a *RAPGEF1 *SNP, rs7034356, we reported here in Table 3. These newly reported and replicated results for *PIGU *and *RAPGEF1*, as well as some as yet unpublished, but replicated GWAS results for other genes we highlight strongly suggest that the availability and analysis of GWAS results across diverse traits may be useful in predicting and supporting functional loci for further biological study.

Creation of a standardized results database allowed us to conduct a functional gene category analysis. The over-representation of cell adhesion genes was strongest among weight- and BMI-associated traits (*P *< 7.1 × 10^-20^). This expands on an earlier report on the over-representation of cell adhesion genes in significant addiction-related GWAS results [17]. The finding was not sensitive to the inclusion of data from specific studies, suggesting either a broad impact of genetic variability in cell adhesion genes on diverse disease etiologies or a systematic bias toward these genes on commercial genotyping arrays. A previous analysis of relative ontology representation of SNPs on major commercial genotyping arrays indicated that genes relating to biological adhesion account for relatively few arrayed genes (~2%) [18]. Current evidence does support roles for cell adhesion molecules in a number of major diseases [19], and notably an ontology-based analysis of the Phase II data from the HapMap project indicated that cell adhesion genes are among the gene groups with the most evidence for recombination in recent human history suggesting potential selective pressures on this group [20]. It is notable that expected gene ontologies were over-represented for specific disease categories (e.g., antigen processing and presentation in rheumatoid arthritis, CNS development and synaptic transmission in ALS, metal ion/sodium/calcium transport in CAD). This finding may be consistent with the hypothesis that multiple loci in related physiological pathways and processes, each with a relatively small magnitude of effect, may make a significant aggregate contribution to genetic risk of complex diseases.

Consistent, widespread standards of reporting and annotation of full disclosure results may facilitate hypothesis-generation and extend discovery that is already occurring from GWAS and their follow-up studies. While GWAS have resulted in the discovery of new and strongly replicated genetic associations relevant to human disease, there continues to be a substantial challenge to discovering meaningful genotype-phenotype associations among a surfeit of data. The typical staged approach to GWAS discovery consists of ranking statistical associations and replication testing in large follow-up sample cohorts; while some "true" associations are found positioned relatively low on the initial p-value ranked list [21]. A recent follow-up meta-analysis across Type II Diabetes GWAS resulted in the identification and replication of additional loci that did not meet genome-wide significant thresholds in any primary GWAS analysis, highlighting the benefit of combining GWAS results from multiple studies [22]. Other studies following initial GWAS data releases have employed pathway-based analyses [23], multilocus association testing [22] and *in silico *comparisons across multiple GWAS for related phenotypes, for example to find SNPs associated with both LDL cholesterol and CAD [25]. As more data become available, further analyses become feasible, including the possibility of using Bayesian inference to weight SNPs with *a priori *evidence for association for use in the analysis of new trait scans [3,26,27]. Weighting of SNPs could be conducted based on a variety of parameters including *a priori *linkage, or functional evidence such as prior gene expression GWAS. Our results (Figure 3) suggest that weighting schemes incorporating gene centricity and tagging of gene regions may be relevant, as previously demonstrated [26].

A growing number of GWAS investigative teams including the Diabetes Genetic Initiative [28], the Wellcome Trust Case Control Consortium [29] and the Framingham Heart Study [30] are leading efforts for the early and wide-spread dissemination of aggregate results from GWAS to enable further scientific research. Informatics initiatives including the National Center for Biotechnology Information's (NCBI) database of Genotype and Phenotype (dbGaP) have a core goal of systematically making available GWAS participant-level data and aggregate results for future analysis [4]. However, our analysis suggests that the extent and quality of further analyses of GWAS results will largely depend upon the extent of SNP results to which researchers have access and the quality of data annotation. We found that a substantial portion of GWAS results are currently unavailable even through an application process, and further that available results are largely presented in a non-uniform manner among disparate databases and web clients, and are often lacking even the most basic gene and SNP annotation. Shifts in SNP-genome positions and SNPids over time and unavailable full SNP lists for some platforms and custom arrays also exacerbate attempts to harmonize results from different studies or genotyping platforms. Further complicating the move to widely distribute aggregate results is the report that the identity of individual research participants may be revealed from large numbers of aggregate genotype-phenotype research results. Estimation methods have been reported, using simple allele frequencies or genotype counts, which make it possible to accurately determine whether specific individuals with known high-density SNP profiles are participants in a complex genomic DNA mixture, such as the case or control groups from publicly available aggregate datasets [31]. In response to this report, access to aggregate genotype data for GWAS studies on dbGaP and other GWAS portals has been removed from public access and made available through controlled access processes requiring the user to receive approval from a data access committee. In total these substantial obstacles to further analysis suggest a need to establish and adopt standards for GWAS reporting.

A previous working group paper suggested criteria for establishing and evaluating GWAS reports and replication, and their report highlights the types of information that would be central to a GWAS data standard [1]. The centralization of GWAS results in a standardized repository containing information similar to that presented in the database here and periodically updated from the literature, could provide a platform for further analysis by the research community with many potential benefits, including functionality for integration with other informatics resources and the ability to iteratively access, search and conduct additional analyses as new scan data becomes available. The establishment of GWAS reporting standards is beyond the scope of this article and requires a dialogue throughout the community. The adoption of MIAME standards for microarray gene expression studies has enabled substantial advances in that field and more systematic bioinformatics analysis of results [32]. In an ideal scenario journals would require authors to make a submission that meets or exceeds a GWAS reporting standard before accepting a paper for publication (Table 5). While the disclosure of genotype results even when appropriately de-identified and subject to other research protections has potential dilemmas ethical and otherwise, the disclosure of association p-values, basic experimental and SNP annotation information may be less problematic. We suggest that in order to also protect the interests of invested researchers who may have ongoing projects following initial GWAS analysis that any minimal standard allow for a lagging time period before the disclosure of full association results.

**Table 5:** Potential criteria for standardized reporting of GWAS results.

Methodological description (array(s), calling algorithm, DNA extraction method, DNA pooling)
Full disclosure of SNP list genotyped, with genomic build and position
Genotyped strand and call rates for each SNP
QC filtering criteria employed
Sample sizes for all analyzed populations and indication of genotyping conducted for each
Sample demographics: gender, mean age, ethnogeographic make-up, geographic coordinates
Specific phenotype description(s)
Description of analytical and statistical procedures applied (genetic models, imputation, etc.)
Full p-values and effects estimates (such as direction of effect with respect to alleles, beta coefficients and standard errors) for association in main GWAS scans, and for meta-analysis if done across scans
List of SNPs with genomic build and position for which replication was attempted
Appropriate measures to mask group and individual identification if data are posted publicly
Contact information for submitters and parties responsible for portions of the GWAS

## Conclusion

We provide a comprehensive open access database of available GWAS results, along with general observations and first analyses. We observed substantial heterogeneity in the amount and type of information currently reported in GWAS articles. After substantial data collection and informatics integration efforts, our first pass analysis across GWAS indicates there may be substantial benefits to centralizing and opening access to GWAS results. We found support for potential pleiotropy of known, replicated loci, as well as the suggestion of new, interesting candidate genes and functional categories that require further validation and study. The creation of an open access resource for GWAS results should encourage and facilitate new genetic and genomic analysis, and provides a potential resource for easier participation in results sharing among interested researchers.

## Competing interests

The authors declare that they have no competing interests.

## Authors' contributions

The project was designed and implemented by ADJ, with significant input from CO. Both ADJ and CO wrote and edited the manuscript.

## Pre-publication history

The pre-publication history for this paper can be accessed here:



## Supplementary Material

Additional file 1 **Summary information on the 118 GWAS studies included in this study.** Information on GWAS including genotyping arrays, phenotype descriptions, discovery and replication samples, analytic strategies, data availability, URLs, publication date and contact information. Data fields are described in detail in Additional file 3. Click here for file

Additional file 2 **56,411 GWAS genotype-phenotype associations and annotation.** The database of significant GWAS associations and additional gene and SNP annotations used in this paper. Data fields are described in detail in Additional file 3. Click here for file

Additional file 3 **Supplemental text.** Supplemental text for the paper providing detailed descriptions of how data fields were ascertained for Additional files 1, 2 and 4, as well as a description of gene ontology analysis, full citation information for 118 GWAS and identification of studies included in disease groups for ontology analysis presented in Additional file 5. Click here for file

Additional file 4 **Microsoft Access 2007 database of 56,411 GWAS genotype-phenotype associations and annotation.** The database of significant GWAS associations and additional gene and SNP annotations used in this paper. Data fields are similar to Additional file 2 and are described in detail in Additional file 3. Click here for file

Additional file 5 **GOminer gene ontology analysis results for GWAS in disease sub-categories.** GO categories significantly enriched among significant disease groupings of GWAS results. Studies included in disease groups are identified in Additional file 3. Click here for file

Additional file 6 **Formatted files for more than 400 GWAS analyses that can be used to upload and browse results in UCSC Genome Browser using Genome Graphs.** This archive file contains Genome Graph files for all GWAS associations contained in Additional files 2 and 4. The files within the archive can be used to visualize GWAS associations described here using UCSC Genome Graphs  at regional, chromosomal and whole genome levels. A file "README.txt" describes file naming conventions. The file "JohnsonODonnell_ALLgwas_graph.txt" contains a single Genome Graph file containing all associations. Click here for file
