# Supplementary material for: An Open Access Database of Genome-wide Association Results
Source: BMC Med Genet. 2009 Jan 22;10:6. doi: 10.1186/1471-2350-10-6 (PMC2639349; doi:10.1186/1471-2350-10-6)
Supplement: Additional file 3 — Supplemental text. Supplemental text for the paper providing detailed descriptions of how data fields were ascertained for Additional files 1, 2 and 4, as well as a description of gene ontology analysis, full citation information for 118 GWAS and identification of studies included in disease groups for ontology analysis presented in Additional file 5. [file 1471-2350-10-6-S3.doc]

Supplemental Text for Johnson and O’Donnell

**Study characteristics for 118 GWAS articles (Additional file 1)**

Additional file 1 provides information on the study design and data from each of the 118 GWAS. The following information was extracted from each study (Additional file 1 columns from left to right):

a) *First author:* study lead author;

b) *Phenotype(s):* description of the primary phenotype(s) studied;

c-l) *Affymetrix* and *Illumina*: Manufacturer and density of genotyping arrays;

m) *Cases*: the number of cases included in the final GWAS discovery analysis—for quantitative traits, this field is blank;

n) *Controls*: the number of controls included in the final GWAS discovery analysis—for quantitative traits, this field is blank;

o) *Total samples analyzed*: the total number of samples included in the final GWAS discovery analysis, representing either the sum of cases and controls or, for quantitative traits, the total number of samples;

p) *Total analyzed SNPs*: the total number of individual SNPs reported to be included in the final GWAS discovery analysis;

q) *Pooled genotyping*: marked ‘X’ if a pooled DNA genotyping strategy was employed in the discovery phase;

r) *Family structure*: marked ‘X’ if the authors reported some family structure in the discovery cohort;

s) *Imputation*: marked ‘X’ if imputation was employed in the discovery phase;

t) *Replication attempted?*: marked ‘X’ if the authors reported an attempt at replication;

u) *Replication cases*: number of cases in the replication sample—for quantitative traits, this field is blank;

v) *Replication controls*: number of controls in replication sample—for quantitative traits, this field is blank;

w) *Total replication samples*: total number of samples used for replication—if the total number does not equal the sum of cases and controls, then an additional replication strategy was employed (e.g., a related quantitative trait was also analyzed);

x) *Replication achieved?*: marked ‘X’ if the authors reported successful replication of at least one marker based on their criteria for replication;

y) *Additional genotyping*: marked ‘X’ if the authors conducted additional genotyping beyond the discovery phase;

z) *Family-based replication*: marked ‘X’ if the authors report that the replication cohort contains family members and family-based analysis is employed;

aa) *In silico comparison*: marked ‘X’ if the authors report using *in silico* comparison strategies to conduct replication (e.g., comparison with other GWAS results directly, through imputation or through LD proxy SNPs);

ab) *Data access model*: “All associations” indicates that statistical p-values for each analyzed SNP are available, “Top associations” indicates that numerous statistical p-values are available, generally p-values below an author-defined threshold (e.g., p<10^-4^); “Few associations” indicates that p-value results for one or relatively few SNPs are made available, or results are restricted to only one or a few loci (e.g., resequencing results); “Top ranked” or “All ranked” indicates rankings were available but without statistical values; and “All genotypes” indicates that authors made available genotypes;

ac) *Publication date*: earliest reported date of publication;

ad) *URL*: description of web-based location of all available results;

ae) *Contact*: corresponding author(s) e-mail addresses.

**Meta-dataset of top associations from 118 GWAS articles (Additional file 2)**

All available associations were collected for each article including from the main text, tables and figures, supplemental material, and additional web sites; however, full association results, when available, were collected from higher density scans (e.g., >200,000 SNPs). All associations with p ≤ 0.001 (after rounding) were retained from raw, discovery scan results. Associations reported from analyses of replication, fine mapping or *a priori* candidate genes were included with p ≤ 0.05. Linkage results were not included. The most statistically significant p-value association per SNP, per article, was then retained along with the following information presented in Additional file 2, in columns from left to right:

1. *Entry ID:* a unique table entry ID # for each association;
2. *First author*: study lead author;
3. *PubMed ID*: PubMed article ID;
4. *Chip:* genotyping array(s) employed;
5. *CaseSamples*: the number of cases included in the final GWAS discovery analysis—for quantitative traits, this field is blank;
6. *ControlSamples*: the number of controls included in the final GWAS discovery analysis—for quantitative traits, this field is blank;
7. *TotalSamples*: the total number of samples included in the final GWAS discovery analysis, either the sum of cases and controls or, for quantitative traits, the total number of samples;
8. *AnalyzedSNPs*: the total number of individual SNPs reported to be included in the final GWAS discovery analysis;
9. *DiscoveryStudyDesign*: GWAS discovery phase study design, includes one or more of: “Association”, “Family-based” if there is family structure in the discovery cohort(s), “Imputation”, and “Experimental/In Vitro”
10. *Addl Study Design*: Additional study design reflects replication or other designs applied, includes one or more of: “Genotyping”, “In silico replication”, “LD inference”, “Family-based” if there is family structure in the replication cohort(s), “Imputation”, “Resequencing”, “Two/Three/Four Stage Design”
11. *RepAtt*: marked ‘X’ if the authors reported attempting replication;
12. *RepSucc*: marked ‘X’ if the authors reported successful replication of at least one marker based on their definition of replication, otherwise, marked ‘N’ for no replication or ‘n/a’ for not attempted (note this does not reflect the individual replication status of any given SNP in Additional file 2);
13. *DataAccess*: Data access model (see description of Additional file 1 above);
14. *URL*: URL or description of virtual location of extended results availability;
15. *DatePub*: publication date (earliest reported);
16. *CorrAuth*: corresponding author(s) e-mail addresses;
17. *Primary_phenotype*: a description of the phenotype(s) studied, not including more detailed analysis subsets (these are described in column U);
18. *SNP_id*: snpID as reported in original GWAS article, or as determined by us if commercial probe IDs or genome positions only were given;
19. *Table_id*: the origin of the most significant association for the SNP within the referenced article;
20. *P_value*: p-value for association;
21. *Secondary_phenotype*: the specific phenotype for which each association was observed (e.g., gender-specific analysis), often, but not always matching the primary phenotype of the article;
22. *Gene(s)*: gene(s) reported for the location of or nearby the associated SNP in the original article;
23. *Imputed_assoc*: marked ‘X’ if the reported association in the row was at least partially derived by imputation;
24. *Pooled?*: marked ‘X’ if a pooled DNA genotyping strategy was employed in the study;
25. *SNPmaps?*: marked “Mapping problem” if the SNP did not clearly map to a single position in human genome Build 36. If there is a mapping problem the rest of the columns will be blank with the possible exception of dbSNP annotation (e.g., allele frequency);
26. *dbSNP128ID_usedInRetrieval*: the dbSNP Build 128 snpID used in the retrieval of current positional information for annotation;
27. *Chr*: chromosome location of SNP in Build 36;

ab) *Position (Build36.2)*: chromosome position of SNP in Build 36;

ac) *InRefGene*: if the SNP is in the transcript boundaries of one or more RefSeq gene they are listed here. Columns AC-AN contain standardized annotation based on SNP position and generated by the GRASP program, integrating information from RefSeq gene positions and UCSC Genome Bioinformatics Known Genes track which includes ~10% additional non-coding genes and alternative isoforms;

ad) *RefGenes <60kb from SNP*: all RefSeq genes with some portion within 60 kb of the SNP position;

ae) *ClosestRefGene*: the closest RefSeq gene boundary to the SNP (including the gene a SNP is contained within);

af) *DistanceFromClosestRefGene*: distance from the SNP to the closest RefSeq gene boundary;

ag) *2ndClosestRefGene*: second closest RefSeq gene boundary to the SNP;

ah) *DistanceFrom2ndRefGene*: distance from the SNP to the second closest RefSeq gene boundary;

ai-an) These columns contain parallel information to columns AC-AH except relative to USCS Known Genes (column headers are the same except RefGene is replaced by KnownGene);

ao) *Strand*: dbSNP build 128 strand information relative to human genome Build 36. Columns AO-AV are all derived from dbSNP build 128 information;

ap) *Alleles*: observed alleles on the strand given in column AO;

aq) *Polymorphism_type*: polymorphism type: “single” (by vast majority), “insertion”, “mixed”;

ar) *Validation*: SNP validation status, one or more of the following: “by-cluster”, “by-frequency”, “by-2hit-2allele”, “by submitter”;

as) *Avg_het*: dbSNP average heterozygosity (averaged across known samples);

at) *Stderr_het*: standard error for the average heterozygosity estimate;

au) *dbSNP_fxn*: dbSNP functional annotation, one or more of the following: “intron”, “locus” (within 2 kb a gene region), “coding-synon”, “coding-nonsynon”, “untranslated”, “unknown”;

av) *SNPaliases*: a list of all other alias SNPids by which the SNP has been known, based on the dbSNP “RsMerge128Arch” table.

**Over-represented gene categories in disease subsets among top GWAS associations (Table 4, Additional file 5)**

We selected subsets of the GWAS meta-data from Additional file 2 according to similar disease phenotypes. We then used standardized RefSeq gene annotations (generated for Additional file 2) to identify all genes with at least one SNP with a significant association within each disease subset. In this manner lists of positively associated genes were created. Gene lists of significant associations for each disease subset were inputted into High-Throughput GOminer^119^. GO data sources selected were *Homo sapiens* (UniProt, TIGR_TGI, LMP), using all evidence codes, enhanced names, cross references, synonyms, and GO biological process terms. Both p-values and FDR constraints were set to ≤ 0.05. Randomizations to derive the FDR thresholds were set to the maximum (n=1,000). We required GO categories to have a minimum of 10 entries to be considered for analysis. Further analytic details of this approach can be found in the paper describing GOminer^119^. Results for all GWAS data regardless of disease category are presented in Table 4. Results for disease subsets are presented in Additional file 5. The disease related subsets included data from the following studies:

Addiction disorders ^6,7,61,106^

Alzheimer’s disease ^13,58,80^

Amyotrophic lateral sclerosis (ALS) ^14,18,87,107^

Blood pressure, Hypertension ^1,45,57,86,109^

Cancer ^8,17,19,20,28,29,40,70,94,95,96,117,118^

Cardiovascular disease ^1,2,27,36,54,55,64,65,71,72,74,83,102^

Crohn’s disease ^1,16,24,60,78,81,115^

Lipid-related traits ^43,44,52,84,86,110,113^

Parkinson’s disease ^25,63^

Rheumatoid Arthritis ^1,76,77,97^

Type II Diabetes ^1,22,32,33,35,66,79,82,86,88,93,99^

Weight/BMI-related traits ^23,37,38,86,89^

Supplemental References

1. **Genome-wide association study of 14,000 cases of seven common diseases and 3,000 shared controls.** *Nature* 2007, **447:** 661-678.

2. Arking DE, Pfeufer A, Post W, Kao WH, Newton-Cheh C, Ikeda M *et al*.: **A common genetic variant in the NOS1 regulator NOS1AP modulates cardiac repolarization.**  *Nat Genet* 2006, **38:** 644-651.

3. Arking DE, Cutler DJ, Brune CW, Teslovich TM, West K, Ikeda M *et al*.: **A common genetic variant in the neurexin superfamily member CNTNAP2 increases familial risk of autism.** *Am J Hum Genet* 2008, **82:** 160-164.

4. Baum AE, Akula N, Cabanero M, Cardona I, Corona W, Klemens B *et al*.: **A genome-wide association study implicates diacylglycerol kinase eta (DGKH) and several other genes in the etiology of bipolar disorder.** *Mol Psychiatry* 2008, **13:** 197-207.

5. Benjamin EJ, Dupuis J, Larson MG, Lunetta KL, Booth SL, Govindaraju DR *et al*.: **Genome-wide association with select biomarker traits in the Framingham Heart Study.** *BMC Med Genet* 2007, **8 Suppl 1:** S11.

6. Berrettini W, Yuan X, Tozzi F, Song K, Francks C, Chilcoat H *et al*.: **Alpha-5/alpha-3 nicotinic receptor subunit alleles increase risk for heavy smoking.** *Mol Psychiatry* 2008, **13:** 368-373.

7. Bierut LJ, Madden PA, Breslau N, Johnson EO, Hatsukami D, Pomerleau OF *et al*.: **Novel genes identified in a high-density genome wide association study for nicotine dependence.** *Hum Mol Genet* 2007, **16:** 24-35.

8. Broderick P, Carvajal-Carmona L, Pittman AM, Webb E, Howarth K, Rowan A *et al*.: **A genome-wide association study shows that common alleles of SMAD7 influence colorectal cancer risk.** *Nat Genet* 2007, **39:** 1315-1317.

9. Buch S, Schafmayer C, Volzke H, Becker C, Franke A, von Eller-Eberstein H *et al*.: **A genome-wide association scan identifies the hepatic cholesterol transporter ABCG8 as a susceptibility factor for human gallstone disease.** *Nat Genet* 2007, **39:** 995-999.

10. Butcher LM, Davis OS, Craig IW, Plomin R: **Genome-wide quantitative trait locus association scan of general cognitive ability using pooled DNA and 500K single nucleotide polymorphism microarrays.** *Genes Brain Behav* 2007.

11. Capon F, Di MP, Szaub J, Prescott NJ, Dunster C, Baumber L *et al*.: **Sequence variants in the genes for the interleukin-23 receptor (IL23R) and its ligand (IL12B) confer protection against psoriasis.** *Hum Genet* 2007, **122:** 201-206.

12. Cervino AC, Tsinoremas NF, Hoffman RW: **A genome-wide study of lupus: preliminary analysis and data release.** *Ann N Y Acad Sci* 2007, **1110:** 131-139.

13. Coon KD, Myers AJ, Craig DW, Webster JA, Pearson JV, Lince DH *et al*.: **A high-density whole-genome association study reveals that APOE is the major susceptibility gene for sporadic late-onset Alzheimer's disease.** *J Clin Psychiatry* 2007, **68:** 613-618.

14. Cronin S, Berger S, Ding J, Schymick JC, Washecka N, Hernandez DG *et al*.: **A genome-wide association study of sporadic ALS in a homogenous Irish population.**  *Hum Mol Genet* 2008, **17:** 768-774.

15. Dewan A, Liu M, Hartman S, Zhang SS, Liu DT, Zhao C *et al*.: **HTRA1 promoter polymorphism in wet age-related macular degeneration.** *Science* 2006, **314:** 989-992.

16. Duerr RH, Taylor KD, Brant SR, Rioux JD, Silverberg MS, Daly MJ *et al*.: **A genome-wide association study identifies IL23R as an inflammatory bowel disease gene.** *Science* 2006, **314:** 1461-1463.

17. Duggan D, Zheng SL, Knowlton M, Benitez D, Dimitrov L, Wiklund F *et al*.: **Two genome-wide association studies of aggressive prostate cancer implicate putative prostate tumor suppressor gene DAB2IP.** *J Natl Cancer Inst* 2007, **99:** 1836-1844.

18. Dunckley T, Huentelman MJ, Craig DW, Pearson JV, Szelinger S, Joshipura K *et al*.: **Whole-genome analysis of sporadic amyotrophic lateral sclerosis.** *N Engl J Med* 2007, **357:** 775-788.

19. Easton DF, Pooley KA, Dunning AM, Pharoah PD, Thompson D, Ballinger DG *et al*.: **Genome-wide association study identifies novel breast cancer susceptibility loci.** *Nature* 2007, **447:** 1087-1093.

20. Eeles RA, Kote-Jarai Z, Giles GG, Olama AA, Guy M, Jugurnauth SK *et al*.: **Multiple newly identified loci associated with prostate cancer susceptibility.** *Nat Genet* 2008, **40:** 316-321.

21. Fellay J, Shianna KV, Ge D, Colombo S, Ledergerber B, Weale M *et al*.: **A whole-genome association study of major determinants for host control of HIV-1.** *Science* 2007, **317:** 944-947.

22. Florez JC, Manning AK, Dupuis J, McAteer J, Irenze K, Gianniny L *et al*.: **A 100K genome-wide association scan for diabetes and related traits in the Framingham Heart Study: replication and integration with other genome-wide datasets.** *Diabetes* 2007, **56:** 3063-3074.

23. Fox CS, Heard-Costa N, Cupples LA, Dupuis J, Vasan RS, Atwood LD: **Genome-wide association to body mass index and waist circumference: the Framingham Heart Study 100K project.** *BMC Med Genet* 2007, **8 Suppl 1:** S18.

24. Franke A, Hampe J, Rosenstiel P, Becker C, Wagner F, Hasler R *et al*.: **Systematic association mapping identifies NELL1 as a novel IBD disease gene.** *PLoS ONE* 2007, **2:** e691.

25. Fung HC, Scholz S, Matarin M, Simon-Sanchez J, Hernandez D, Britton A *et al*.: **Genome-wide genotyping in Parkinson's disease and neurologically normal controls: first stage analysis and public release of data.** *Lancet Neurol* 2006, **5:** 911-916.

26. Gottlieb DJ, O'Connor GT, Wilk JB: **Genome-wide association of sleep and circadian phenotypes.** *BMC Med Genet* 2007, **8 Suppl 1:** S9.

27. Gudbjartsson DF, Arnar DO, Helgadottir A, Gretarsdottir S, Holm H, Sigurdsson A *et al*.: **Variants conferring risk of atrial fibrillation on chromosome 4q25.**  *Nature* 2007, **448:** 353-357.

28. Gudmundsson J, Sulem P, Steinthorsdottir V, Bergthorsson JT, Thorleifsson G, Manolescu A *et al*.: **Two variants on chromosome 17 confer prostate cancer risk, and the one in TCF2 protects against type 2 diabetes.** *Nat Genet* 2007, **39:** 977-983.

29. Gudmundsson J, Sulem P, Manolescu A, Amundadottir LT, Gudbjartsson D, Helgason A *et al*.: **Genome-wide association study identifies a second prostate cancer susceptibility variant at 8q24.** *Nat Genet* 2007, **39:** 631-637.

30. Hafler DA, Compston A, Sawcer S, Lander ES, Daly MJ, De Jager PL *et al*.: **Risk alleles for multiple sclerosis identified by a genomewide study.** *N Engl J Med* 2007, **357:** 851-862.

31. Hakonarson H, Grant SF, Bradfield JP, Marchand L, Kim CE, Glessner JT *et al*.: **A genome-wide association study identifies KIAA0350 as a type 1 diabetes gene.** *Nature* 2007, **448:** 591-594.

32. Hanson RL, Bogardus C, Duggan D, Kobes S, Knowlton M, Infante AM *et al*.: **A search for variants associated with young-onset type 2 diabetes in American Indians in a 100K genotyping array.** *Diabetes* 2007, **56:** 3045-3052.

33. Hanson RL, Craig DW, Millis MP, Yeatts KA, Kobes S, Pearson JV *et al*.: **Identification of PVT1 as a candidate gene for end-stage renal disease in type 2 diabetes using a pooling-based genome-wide single nucleotide polymorphism association study.** *Diabetes* 2007, **56:** 975-983.

34. Harley JB, arcon-Riquelme ME, Criswell LA, Jacob CO, Kimberly RP, Moser KL *et al*.: **Genome-wide association scan in women with systemic lupus erythematosus identifies susceptibility variants in ITGAM, PXK, KIAA1542 and other loci.** *Nat Genet* 2008, **40:** 204-210.

35. Hayes MG, Pluzhnikov A, Miyake K, Sun Y, Ng MC, Roe CA *et al*.: **Identification of type 2 diabetes genes in Mexican Americans through genome-wide association studies.** *Diabetes* 2007, **56:** 3033-3044.

36. Helgadottir A, Thorleifsson G, Manolescu A, Gretarsdottir S, Blondal T, Jonasdottir A *et al*.: **A common variant on chromosome 9p21 affects the risk of myocardial infarction.** *Science* 2007, **316:** 1491-1493.

37. Herbert A, Gerry NP, McQueen MB, Heid IM, Pfeufer A, Illig T *et al*.: **A common genetic variant is associated with adult and childhood obesity.** *Science* 2006, **312:** 279-283.

38. Hinney A, Nguyen TT, Scherag A, Friedel S, Bronner G, Muller TD *et al*.: **Genome Wide Association (GWA) Study for Early Onset Extreme Obesity Supports the Role of Fat Mass and Obesity Associated Gene (FTO) Variants.** *PLoS ONE* 2007, **2:** e1361.

39. Huentelman MJ, Papassotiropoulos A, Craig DW, Hoerndli FJ, Pearson JV, Huynh KD *et al*.: **Calmodulin-binding transcription activator 1 (CAMTA1) alleles predispose human episodic memory performance.** *Hum Mol Genet* 2007, **16:** 1469-1477.

40. Hunter DJ, Kraft P, Jacobs KB, Cox DG, Yeager M, Hankinson SE *et al*.: **A genome-wide association study identifies alleles in FGFR2 associated with risk of sporadic postmenopausal breast cancer.** *Nat Genet* 2007, **39:** 870-874.

41. Hwang SJ, Yang Q, Meigs JB, Pearce EN, Fox CS: **A genome-wide association for kidney function and endocrine-related traits in the NHLBI's Framingham Heart Study.** *BMC Med Genet* 2007, **8 Suppl 1:** S10.

42. Kamatani Y, Matsuda K, Ohishi T, Ohtsubo S, Yamazaki K, Iida A *et al*.: **Identification of a significant association of a single nucleotide polymorphism in TNXB with systemic lupus erythematosus in a Japanese population.** *J Hum Genet* 2008, **53:** 64-73.

43. Kathiresan S, Manning AK, Demissie S, D'Agostino RB, Surti A, Guiducci C *et al*.: **A genome-wide association study for blood lipid phenotypes in the Framingham Heart Study.** *BMC Med Genet* 2007, **8 Suppl 1:** S17.

44. Kathiresan S, Melander O, Guiducci C, Surti A, Burtt NP, Rieder MJ *et al*.: **Six new loci associated with blood low-density lipoprotein cholesterol, high-density lipoprotein cholesterol or triglycerides in humans.** *Nat Genet* 2008, **40:** 189-197.

45. Kato N, Miyata T, Tabara Y, Katsuya T, Yanai K, Hanada H *et al*.: **High-density association study and nomination of susceptibility genes for hypertension in the Japanese National Project.** *Hum Mol Genet* 2008, **17:** 617-627.

46. Kawase T, Nannya Y, Torikai H, Yamamoto G, Onizuka M, Morishima S *et al*.: **Identification of human minor histocompatibility antigens based on genetic association with highly parallel genotyping of pooled DNA.** *Blood* 2008, **111:** 3286-3294.

47. Kayser M, Liu F, Janssens AC, Rivadeneira F, Lao O, van DK *et al*.: **Three genome-wide association studies and a linkage analysis identify HERC2 as a human iris color gene.** *Am J Hum Genet* 2008, **82:** 411-423.

48. Kiel DP, Demissie S, Dupuis J, Lunetta KL, Murabito JM, Karasik D: **Genome-wide association with bone mass and geometry in the Framingham Heart Study.** *BMC Med Genet* 2007, **8 Suppl 1:** S14.

49. Kindmark A, Jawaid A, Harbron CG, Barratt BJ, Bengtsson OF, Andersson TB *et al*.: **Genome-wide pharmacogenetic investigation of a hepatic adverse event without clinical signs of immunopathology suggests an underlying immune pathogenesis.**  *Pharmacogenomics J* 2007.

50. Klein RJ, Zeiss C, Chew EY, Tsai JY, Sackler RS, Haynes C *et al*.: **Complement factor H polymorphism in age-related macular degeneration.** *Science* 2005, **308:** 385-389.

51. Kong A, Thorleifsson G, Stefansson H, Masson G, Helgason A, Gudbjartsson DF *et al*.: **Sequence variants in the RNF212 gene associate with genome-wide recombination rate.** *Science* 2008, **319:** 1398-1401.

52. Kooner JS, Chambers JC, guilar-Salinas CA, Hinds DA, Hyde CL, Warnes GR *et al*.: **Genome-wide scan identifies variation in MLXIPL associated with plasma triglycerides.** *Nat Genet* 2008, **40:** 149-151.

53. Kozyrev SV, Abelson AK, Wojcik J, Zaghlool A, Linga Reddy MV, Sanchez E *et al*.: **Functional variants in the B-cell gene BANK1 are associated with systemic lupus erythematosus.** *Nat Genet* 2008, **40:** 211-216.

54. Kubo M, Hata J, Ninomiya T, Matsuda K, Yonemoto K, Nakano T *et al*.: **A nonsynonymous SNP in PRKCH (protein kinase C eta) increases the risk of cerebral infarction.** *Nat Genet* 2007, **39:** 212-217.

55. Larson MG, Atwood LD, Benjamin EJ, Cupples LA, D'Agostino RB, Sr., Fox CS *et al*.: **Framingham Heart Study 100K project: genome-wide associations for cardiovascular disease outcomes.** *BMC Med Genet* 2007, **8 Suppl 1:** S5.

56. Lencz T, Morgan TV, Athanasiou M, Dain B, Reed CR, Kane JM *et al*.: **Converging evidence for a pseudoautosomal cytokine receptor gene locus in schizophrenia.**  *Mol Psychiatry* 2007, **12:** 572-580.

57. Levy D, Larson MG, Benjamin EJ, Newton-Cheh C, Wang TJ, Hwang SJ *et al*.: **Framingham Heart Study 100K Project: genome-wide associations for blood pressure and arterial stiffness.** *BMC Med Genet* 2007, **8 Suppl 1:** S3.

58. Li H, Wetten S, Li L, St Jean PL, Upmanyu R, Surh L *et al*.: **Candidate single-nucleotide polymorphisms from a genomewide association study of Alzheimer disease.** *Arch Neurol* 2008, **65:** 45-53.

59. Li S, Sanna S, Maschio A, Busonero F, Usala G, Mulas A *et al*.: **The GLUT9 Gene Is Associated with Serum Uric Acid Levels in Sardinia and Chianti Cohorts.** *PLoS Genet* 2007, **3:** e194.

60. Libioulle C, Louis E, Hansoul S, Sandor C, Farnir F, Franchimont D *et al*.: **Novel Crohn disease locus identified by genome-wide association maps to a gene desert on 5p13.1 and modulates expression of PTGER4.** *PLoS Genet* 2007, **3:** e58.

61. Liu QR, Drgon T, Johnson C, Walther D, Hess J, Uhl GR: **Addiction molecular genetics: 639,401 SNP whole genome association identifies many "cell adhesion" genes.** *Am J Med Genet B Neuropsychiatr Genet* 2006, **141:** 918-925.

62. Lunetta KL, D'Agostino RB, Sr., Karasik D, Benjamin EJ, Guo CY, Govindaraju R *et al*.: **Genetic correlates of longevity and selected age-related phenotypes: a genome-wide association study in the Framingham Study.** *BMC Med Genet* 2007, **8 Suppl 1:** S13.

63. Maraganore DM, de AM, Lesnick TG, Strain KJ, Farrer MJ, Rocca WA *et al*.: **High-resolution whole-genome association study of Parkinson disease.** *Am J Hum Genet* 2005, **77:** 685-693.

64. Matarin M, Brown WM, Scholz S, Simon-Sanchez J, Fung HC, Hernandez D *et al*.: **A genome-wide genotyping study in patients with ischaemic stroke: initial analysis and data release.** *Lancet Neurol* 2007, **6:** 414-420.

65. McPherson R, Pertsemlidis A, Kavaslar N, Stewart A, Roberts R, Cox DR *et al*.: **A common allele on chromosome 9 associated with coronary heart disease.** *Science* 2007, **316:** 1488-1491.

66. Meigs JB, Manning AK, Fox CS, Florez JC, Liu C, Cupples LA *et al*.: **Genome-wide association with diabetes-related traits in the Framingham Heart Study.** *BMC Med Genet* 2007, **8 Suppl 1:** S16.

67. Melquist S, Craig DW, Huentelman MJ, Crook R, Pearson JV, Baker M *et al*.: **Identification of a novel risk locus for progressive supranuclear palsy by a pooled genomewide scan of 500,288 single-nucleotide polymorphisms.** *Am J Hum Genet* 2007, **80:** 769-778.

68. Menzel S, Garner C, Gut I, Matsuda F, Yamaguchi M, Heath S *et al*.: **A QTL influencing F cell production maps to a gene encoding a zinc-finger protein on chromosome 2p15.** *Nat Genet* 2007, **39:** 1197-1199.

69. Moffatt MF, Kabesch M, Liang L, Dixon AL, Strachan D, Heath S *et al*.: **Genetic variants regulating ORMDL3 expression contribute to the risk of childhood asthma.** *Nature* 2007, **448:** 470-473.

70. Murabito JM, Rosenberg CL, Finger D, Kreger BE, Levy D, Splansky GL *et al*.: **A genome-wide association study of breast and prostate cancer in the NHLBI's Framingham Heart Study.** *BMC Med Genet* 2007, **8 Suppl 1:** S6.

71. Newton-Cheh C, Guo CY, Wang TJ, O'Donnell CJ, Levy D, Larson MG: **Genome-wide association study of electrocardiographic and heart rate variability traits: the Framingham Heart Study.** *BMC Med Genet* 2007, **8 Suppl 1:** S7.

72. O'Donnell CJ, Cupples LA, D'Agostino RB, Fox CS, Hoffmann U, Hwang SJ *et al*.: **Genome-wide association study for subclinical atherosclerosis in major arterial territories in the NHLBI's Framingham Heart Study.** *BMC Med Genet* 2007, **8 Suppl 1:** S4.

73. Obara W, Iida A, Suzuki Y, Tanaka T, Akiyama F, Maeda S *et al*.: **Association of single-nucleotide polymorphisms in the polymeric immunoglobulin receptor gene with immunoglobulin A nephropathy (IgAN) in Japanese patients.** *J Hum Genet* 2003, **48:** 293-299.

74. Ozaki K, Ohnishi Y, Iida A, Sekine A, Yamada R, Tsunoda T *et al*.: **Functional SNPs in the lymphotoxin-alpha gene that are associated with susceptibility to myocardial infarction.** *Nat Genet* 2002, **32:** 650-654.

75. Papassotiropoulos A, Stephan DA, Huentelman MJ, Hoerndli FJ, Craig DW, Pearson JV *et al*.: **Common Kibra alleles are associated with human memory performance.** *Science* 2006, **314:** 475-478.

76. Plenge RM, Seielstad M, Padyukov L, Lee AT, Remmers EF, Ding B *et al*.: **TRAF1-C5 as a risk locus for rheumatoid arthritis--a genomewide study.** *N Engl J Med* 2007, **357:** 1199-1209.

77. Plenge RM, Cotsapas C, Davies L, Price AL, de Bakker PI, Maller J *et al*.: **Two independent alleles at 6q23 associated with risk of rheumatoid arthritis.** *Nat Genet* 2007, **39:** 1477-1482.

78. Raelson JV, Little RD, Ruether A, Fournier H, Paquin B, Van EP *et al*.: **Genome-wide association study for Crohn's disease in the Quebec Founder Population identifies multiple validated disease loci.** *Proc Natl Acad Sci U S A* 2007, **104:** 14747-14752.

79. Rampersaud E, Damcott CM, Fu M, Shen H, McArdle P, Shi X *et al*.: **Identification of novel candidate genes for type 2 diabetes from a genome-wide association scan in the Old Order Amish: evidence for replication from diabetes-related quantitative traits and from independent populations.** *Diabetes* 2007, **56:** 3053-3062.

80. Reiman EM, Webster JA, Myers AJ, Hardy J, Dunckley T, Zismann VL *et al*.: **GAB2 alleles modify Alzheimer's risk in APOE epsilon4 carriers.** *Neuron* 2007, **54:** 713-720.

81. Rioux JD, Xavier RJ, Taylor KD, Silverberg MS, Goyette P, Huett A *et al*.: **Genome-wide association study identifies new susceptibility loci for Crohn disease and implicates autophagy in disease pathogenesis.** *Nat Genet* 2007, **39:** 596-604.

82. Salonen JT, Uimari P, Aalto JM, Pirskanen M, Kaikkonen J, Todorova B *et al*.: **Type 2 diabetes whole-genome association study in four populations: the DiaGen consortium.** *Am J Hum Genet* 2007, **81:** 338-345.

83. Samani NJ, Erdmann J, Hall AS, Hengstenberg C, Mangino M, Mayer B *et al*.: **Genomewide association analysis of coronary artery disease.** *N Engl J Med* 2007, **357:** 443-453.

84. Sandhu MS, Waterworth DM, Debenham SL, Wheeler E, Papadakis K, Zhao JH *et al*.: **LDL-cholesterol concentrations: a genome-wide association study.** *Lancet* 2008, **371:** 483-491.

85. Sanna S, Jackson AU, Nagaraja R, Willer CJ, Chen WM, Bonnycastle LL *et al*.: **Common variants in the GDF5-UQCC region are associated with variation in human height.** *Nat Genet* 2008, **40:** 198-203.

86. Saxena R, Voight BF, Lyssenko V, Burtt NP, de Bakker PI, Chen H *et al*.: **Genome-wide association analysis identifies loci for type 2 diabetes and triglyceride levels.** *Science* 2007, **316:** 1331-1336.

87. Schymick JC, Scholz SW, Fung HC, Britton A, Arepalli S, Gibbs JR *et al*.: **Genome-wide genotyping in amyotrophic lateral sclerosis and neurologically normal controls: first stage analysis and public release of data.** *Lancet Neurol* 2007, **6:** 322-328.

88. Scott LJ, Mohlke KL, Bonnycastle LL, Willer CJ, Li Y, Duren WL *et al*.: **A genome-wide association study of type 2 diabetes in Finns detects multiple susceptibility variants.** *Science* 2007, **316:** 1341-1345.

89. Scuteri A, Sanna S, Chen WM, Uda M, Albai G, Strait J *et al*.: **Genome-Wide Association Scan Shows Genetic Variants in the FTO Gene Are Associated with Obesity-Related Traits.** *PLoS Genet* 2007, **3:** e115.

90. Seshadri S, DeStefano AL, Au R, Massaro JM, Beiser AS, Kelly-Hayes M *et al*.: **Genetic correlates of brain aging on MRI and cognitive test measures: a genome-wide association and linkage analysis in the Framingham Study.** *BMC Med Genet* 2007, **8 Suppl 1:** S15.

91. Shifman S, Bhomra A, Smiley S, Wray NR, James MR, Martin NG *et al*.: **A whole genome association study of neuroticism using DNA pooling.** *Mol Psychiatry* 2008, **13:** 302-312.

92. Shifman S, Johannesson M, Bronstein M, Chen SX, Collier DA, Craddock NJ *et al*.: **Genome-Wide Association Identifies a Common Variant in the Reelin Gene That Increases the Risk of Schizophrenia Only in Women.** *PLoS Genet* 2008, **4:** e28.

93. Sladek R, Rocheleau G, Rung J, Dina C, Shen L, Serre D *et al*.: **A genome-wide association study identifies novel risk loci for type 2 diabetes.** *Nature* 2007, **445:** 881-885.

94. Spinola M, Meyer P, Kammerer S, Falvella FS, Boettger MB, Hoyal CR *et al*.: **Association of the PDCD5 locus with lung cancer risk and prognosis in smokers.** *J Clin Oncol* 2006, **24:** 1672-1678.

95. Spinola M, Leoni VP, Galvan A, Korsching E, Conti B, Pastorino U *et al*.: **Genome-wide single nucleotide polymorphism analysis of lung cancer risk detects the KLF6 gene.** *Cancer Lett* 2007, **251:** 311-316.

96. Stacey SN, Manolescu A, Sulem P, Rafnar T, Gudmundsson J, Gudjonsson SA *et al*.: **Common variants on chromosomes 2q35 and 16q12 confer susceptibility to estrogen receptor-positive breast cancer.** *Nat Genet* 2007, **39:** 865-869.

97. Steer S, Abkevich V, Gutin A, Cordell HJ, Gendall KL, Merriman ME *et al*.: **Genomic DNA pooling for whole-genome association scans in complex disease: empirical demonstration of efficacy in rheumatoid arthritis.** *Genes Immun* 2007, **8:** 57-68.

98. Stefansson H, Rye DB, Hicks A, Petursson H, Ingason A, Thorgeirsson TE *et al*.: **A genetic risk factor for periodic limb movements in sleep.** *N Engl J Med* 2007, **357:** 639-647.

99. Steinthorsdottir V, Thorleifsson G, Reynisdottir I, Benediktsson R, Jonsdottir T, Walters GB *et al*.: **A variant in CDKAL1 influences insulin response and risk of type 2 diabetes.** *Nat Genet* 2007, **39:** 770-775.

100. Stokowski RP, Pant PV, Dadd T, Fereday A, Hinds DA, Jarman C *et al*.: **A genomewide association study of skin pigmentation in a South Asian population.** *Am J Hum Genet* 2007, **81:** 1119-1132.

101. Sulem P, Gudbjartsson DF, Stacey SN, Helgason A, Rafnar T, Magnusson KP *et al*.: **Genetic determinants of hair, eye and skin pigmentation in Europeans.** *Nat Genet* 2007, **39:** 1443-1452.

102. Suzuki S, Yoshimura M, Nakayama M, Abe K, Yamamuro M, Nagayoshi Y *et al*.: **A novel genetic marker for coronary spasm in women from a genome-wide single nucleotide polymorphism analysis.** *Pharmacogenet Genomics* 2007, **17:** 919-930.

103. Thorleifsson G, Magnusson KP, Sulem P, Walters GB, Gudbjartsson DF, Stefansson H *et al*.: **Common sequence variants in the LOXL1 gene confer susceptibility to exfoliation glaucoma.** *Science* 2007, **317:** 1397-1400.

104. Tomlinson I, Webb E, Carvajal-Carmona L, Broderick P, Kemp Z, Spain S *et al*.: **A genome-wide association scan of tag SNPs identifies a susceptibility variant for colorectal cancer at 8q24.21.** *Nat Genet* 2007, **39:** 984-988.

105. Uda M, Galanello R, Sanna S, Lettre G, Sankaran VG, Chen W *et al*.: **Genome-wide association study shows BCL11A associated with persistent fetal hemoglobin and amelioration of the phenotype of beta-thalassemia.** *Proc Natl Acad Sci U S A* 2008, **105:** 1620-1625.

106. Uhl GR, Liu QR, Drgon T, Johnson C, Walther D, Rose JE: **Molecular genetics of nicotine dependence and abstinence: whole genome association using 520,000 SNPs.** *BMC Genet* 2007, **8:** 10.

107. van Es MA, Van Vught PW, Blauw HM, Franke L, Saris CG, Andersen PM *et al*.: **ITPR2 as a susceptibility gene in sporadic amyotrophic lateral sclerosis: a genome-wide association study.** *Lancet Neurol* 2007, **6:** 869-877.

108. van Heel DA, Franke L, Hunt KA, Gwilliam R, Zhernakova A, Inouye M *et al*.: **A genome-wide association study for celiac disease identifies risk variants in the region harboring IL2 and IL21.** *Nat Genet* 2007, **39:** 827-829.

109. Vasan RS, Larson MG, Aragam J, Wang TJ, Mitchell GF, Kathiresan S *et al*.: **Genome-wide association of echocardiographic dimensions, brachial artery endothelial function and treadmill exercise responses in the Framingham Heart Study.** *BMC Med Genet* 2007, **8 Suppl 1:** S2.

110. Wallace C, Newhouse SJ, Braund P, Zhang F, Tobin M, Falchi M *et al*.: **Genome-wide association study identifies genes for biomarkers of cardiovascular disease: serum urate and dyslipidemia.** *Am J Hum Genet* 2008, **82:** 139-149.

111. Weedon MN, Lettre G, Freathy RM, Lindgren CM, Voight BF, Perry JR *et al*.: **A common variant of HMGA2 is associated with adult and childhood height in the general population.** *Nat Genet* 2007, **39:** 1245-1250.

112. Wilk JB, Walter RE, Laramie JM, Gottlieb DJ, O'Connor GT: **Framingham Heart Study genome-wide association: results for pulmonary function measures.** *BMC Med Genet* 2007, **8 Suppl 1:** S8.

113. Willer CJ, Sanna S, Jackson AU, Scuteri A, Bonnycastle LL, Clarke R *et al*.: **Newly identified loci that influence lipid concentrations and risk of coronary artery disease.** *Nat Genet* 2008, **40:** 161-169.

114. Winkelmann J, Schormair B, Lichtner P, Ripke S, Xiong L, Jalilzadeh S *et al*.: **Genome-wide association study of restless legs syndrome identifies common variants in three genomic regions.** *Nat Genet* 2007, **39:** 1000-1006.

115. Yamazaki K, McGovern D, Ragoussis J, Paolucci M, Butler H, Jewell D *et al*.: **Single nucleotide polymorphisms in TNFSF15 confer susceptibility to Crohn's disease.** *Hum Mol Genet* 2005, **14:** 3499-3506.

116. Yang Q, Kathiresan S, Lin JP, Tofler GH, O'Donnell CJ: **Genome-wide association and linkage analyses of hemostatic factors and hematological phenotypes in the Framingham Heart Study.** *BMC Med Genet* 2007, **8 Suppl 1:** S12.

117. Yeager M, Orr N, Hayes RB, Jacobs KB, Kraft P, Wacholder S *et al*.: **Genome-wide association study of prostate cancer identifies a second risk locus at 8q24.** *Nat Genet* 2007, **39:** 645-649.

118. Zanke BW, Greenwood CM, Rangrej J, Kustra R, Tenesa A, Farrington SM *et al*.: **Genome-wide association scan identifies a colorectal cancer susceptibility locus on chromosome 8q24.** *Nat Genet* 2007, **39:** 989-994.

119. Zeeberg BR, Qin H, Narasimhan S, Sunshine M, Cao H, Kane DW *et al*.: **High-Throughput GoMiner, an 'industrial-strength' integrative gene ontology tool for interpretation of multiple-microarray experiments, with application to studies of Common Variable Immune Deficiency (CVID).** *BMC Bioinformatics* 2005, **6:** 168.
